# Diagnostic accuracy of radiolabelled-WBC scintigraphy in patients with antibiotic therapy

**DOI:** 10.1007/s00259-026-07855-w

**Published:** 2026-04-29

**Authors:** Chiara Lauri, Giuseppe Campagna, Roberta Ottaviani, Mariano Pontico, Michela Varani, Valeria Bentivoglio, Simone Tetti, Walter Davide Vella, Miriam Lichtner, Alberto Signore

**Affiliations:** 1 Department of Medical-Surgical Sciences and Translational Medicine, Unit of Nuclear Medicine, Faculty of Medicine and Psychology, “Sapienza” University of Rome, Rome, Italy; 2 Nuclear Medicine Unit, University Hospital Sant’Andrea, Rome, Italy; 3 Department of Neurosciences, Mental Health and Sense Organs – NESMOS, Unit of Infective Diseases, Faculty of Medicine and Psychology, “Sapienza” University of Rome, Rome, Italy

**Keywords:** White blood cell scintigraphy, Infection imaging, Osteomyelitis, Cardiovascular infection, Soft tissue infection

## Abstract

**Purpose:**

The aim of this retrospective study was to assess to what extent antibiotic therapy impacts the accuracy of ⁹⁹ᵐTc-HMPAO labelled white blood cell (WBC) scintigraphy.

**Methods:**

We analyzed 1016 patients who performed WBC scintigraphy, according to EANM guidelines, between May 2014 and September 2025. Only patients with a definite final diagnosis were enrolled (484 patients). Patients were stratified into six groups based on antibiotic therapy status at the time of examination: 1) ongoing antibiotic therapy, 2) discontinued for ≤ 7 days, 3) discontinued for 8–15 days, 4) discontinued for 16–30 days, 5) discontinued for > 30 days or 6) without antibiotic therapy.

**Results:**

Overall, we observed a sensitivity of 66%, a specificity of 97% and 86% of accuracy. Sensitivity was markedly reduced in patients receiving antibiotics at the time of testing (30%) and increased according to therapy withdrawal time, reaching 100% in groups 4 and 5. A similar trend was observed for accuracy and NPV. Since we found no difference between groups 1, 2 and 3, these were grouped together and compared to groups 4, 5 and 6 grouped together. The best performance, in terms of sensitivity, specificity, accuracy and NPV was achieved in patients who were not receiving treatment or who had stopped treatment for more than 15 days (*p* < 0.0001).

**Conclusion:**

Results of this study indicate that the accuracy of radiolabelled WBC scintigraphy is influenced by ongoing antibiotic therapy. Based on our results, 15 days of suspension are advisable to perform WBC scintigraphy.

**Supplementary Information:**

The online version contains supplementary material available at 10.1007/s00259-026-07855-w.

## Introduction

Scintigraphy with radiolabeled white blood cells (WBC), with **⁹⁹**ᵐTc-HMPAO or ^111^In-oxine, is one of the reference methods for imaging infections. Its high specificity derives from the direct tracking of granulocyte migration and homing in active infectious sites and can be further increased by SPECT/CT which allows correct evaluation of disease’s extent [1]. The guidelines of the European Association of Nuclear Medicine (EANM) define its indications, labeling protocols, acquisition and interpretation criteria, especially for osteomyelitis, infections of orthopedic or vascular prostheses, diabetic foot, cardiovascular, fever of unknown origin and soft tissue infections. These guidelines aim to ensure methodological uniformity and optimize the diagnostic reproducibility of the technique [1–9].

In parallel, the clinical appropriateness criteria developed by the American Society of Nuclear Medicine and Molecular Imaging (SNMMI) define when nuclear imaging with labeled leukocytes is indicated based on pre-test probability, clinical question and available diagnostic alternatives [10].

In literature, WBC scan has reported high sensitivity and specificity values ​​for many clinical indications, especially when standardized interpretation criteria and correct acquisition protocols are applied [11–13]. However, despite the methodological robustness of WBC scan, several variables can potentially influence its diagnostic accuracy, including the timing of the test with respect to any surgical intervention and, above all, the presence of ongoing antibiotic therapy.

The possibility that antimicrobial treatment interferes with the diagnostic performance of labelled leukocyte scintigraphy has always been a matter of debate.

Two main pathophysiological mechanisms have been proposed through which antibiotic therapy could reduce the sensitivity of labeled leukocyte scintigraphy. First, the antibacterial action reduces the microbial load and the production of chemo-attractive mediators, attenuating the gradient responsible for neutrophil recruitment. Second, some antibiotics can exert a direct effect on leukocytes, modulating functions such as chemotaxis and cell motility, resulting in reduced migration toward the infectious site [14–19].

This issue is highly relevant from a clinical perspective, as many patients start empirical antibiotic therapy before the infection site is identified, with difficulty of interpreting WBC scan at diagnosis or at follow-up. Available literature is heterogeneous, focused on different clinical indications [16–20] and, most important, it does not include prospective, controlled studies directly comparing patients receiving and not receiving antibiotic therapy. Moreover, duration of therapy is rarely reported in these studies thus limiting the possibility of identifying clear temporal cutoffs.

The variability of results reported in the literature, combined with the lack of prospective, controlled studies, requires further investigation into the potential impact of antibiotic therapy on diagnostic accuracy of labelled leukocyte scintigraphy in its main clinical indications.

Attempting to address this unmet clinical need, the primary aims of this retrospective study were to assess whether antimicrobial therapy negatively affects the accuracy of radiolabelled WBC scintigraphy and to identify a potential antibiotic-free interval that allows radiolabeled leukocyte scintigraphy retain its own high diagnostic performance.

## Materials and methods

### Study design

This observational, single-center study was conducted through a retrospective analysis of clinical, microbiological and imaging data from patients undergoing radiolabelled WBC scintigraphy. Data collection and analysis were conducted in compliance with current regulations regarding privacy and clinical research.

### Setting

In our study, we retrospectively reviewed the clinical and scintigraphic data of patients who underwent radiolabeled WBC scintigraphy at the Nuclear Medicine Unit of the Sant'Andrea University Hospital in Rome between May 2014 and September 2025 (136 months).

Information on the presence/absence of antibiotic treatment and any interruption interval before the examination were recorded at the time of the scan and included in the final report.

### Participants

Patients who met the following criteria were included in the study:

- Scintigraphy with radiolabelled WBC performed at our Unit during the study period;
- Clinical suspicion of bone/joint, cardiovascular or soft tissue infection;
- Adequate clinical and/or microbiological follow-up (≥ 12 months for clinical follow-up), suitable to assess whether the infection was present or not;
- Reliable information on antibiotic therapy status, including the presence/absence of treatment and the interval between treatment discontinuation prior to the examination;
- Complete and evaluable planar acquisitions according to EANM recommendations (early, delayed and late), regardless of whether SPECT/CT was performed.

Patients who did not meet the above-mentioned inclusion criteria or patients with scintigraphic findings classified as “equivocal” or uninterpretable, regardless of the availability of SPECT/CT were excluded from the study.

White blood cell count, erythrocyte sedimentation rate (ESR), C-reactive protein (CRP) and procalcitonin (PCT) as well as the time of beginning of symptoms and time from surgery, were also collected and analyzed when such information was included in the final report of WBC scan.

Based on antibiotic therapy status at the time of WBC scintigraphy, the cohort was divided into six groups:

1. Ongoing antibiotic therapy;
2. Therapy discontinued within 7 days;
3. Therapy discontinued between 8 and 15 days;
4. Therapy discontinued between 16 and 30 days;
5. Therapy discontinued for more than 30 days;
6. No antibiotic therapy at the time of the examination.

All patients had previously signed informed consent for the labelled leukocyte scintigraphy, as required by current regulations. The study protocol was approved by the competent Ethics Committee (Ethical Committee approval no. 0348 of "Comitato Etico Territoriale Lazio Area 1").

### ^99m^Tc-HMPAO-WBC scintigraphy and image interpretation

Autologous WBC labelling with ^99m^Tc-HMPAO was performed following the EANM procedural guidelines, using a dedicated commercial kit (Leukokit®, CellTech, Italy) and with the prescribed quality controls [1].

Image acquisition was performed using a dual-head gamma camera (SkyLight, Philips or Symbia, Siemens) equipped with low-energy, high-resolution (LEHR) collimators, setting an energy peak at 140 keV with a 20% window. Anterior and posterior planar images were obtained with a 256 × 256 matrix. Based on the clinical indication, images of the region of interest and whole-body images were acquired, possibly supplemented by SPECT/CT scans to improve anatomical localization and to better define the extent of the infective process.

The image acquisition protocol included three series of planar images:

- Early: within 30 min post-injection, with acquisition times of 100 s;
- Delayed: 3 h after the first acquisition, with acquisition times of 141 s;
- Late: 20 h after the first acquisition, with acquisition times of 1007 s.

In vivo quality control was performed on the early images by assessing physiological biodistribution in lungs, liver and spleen.

SPECT/CT of the region of interest was performed at late images with a dedicated hybrid system using a 128 × 128 matrix, 360° rotation, 6° steps, an acquisition of 30 s/frame. The following low-dose CT parameters were applied: A 140 kV, 90 mA, 0.8/s tube rotation, and 5 mm thickness.

Image interpretation was performed by two experienced nuclear medicine physicians (AS, CL).

All images acquired at different time points were displayed with the same intensity scale in absolute counts, to ensure temporal comparability and avoid bias due to manual adjustment of the intensity of the activity scale by the operator.

Based on EANM guidelines, the scan was interpreted:

- positive for infection, when the intensity or extent of uptake in the region of interest increased over time;
- negative for injection, when there was no uptake or a reduction in signal in late images compared to delayed images;
- equivocal, when the uptake behavior was not clearly interpretable.

### Final diagnosis

To calculate diagnostic performance of radiolabelled WBC scintigraphy, final diagnosis was achieved by the availability of microbiological data provided by the patients at the time of the examination and included in the final report or, when not available, by clinical follow-up through telephone interview conducted 12–24 months after scintigraphic assessment. Telephone consultation was based on asking the patients to provide information on their clinical status, laboratory data or other imaging examination collected during follow-up. Patients were excluded if they were unable to provide complete and reliable information or if patient was unable to furnish clinical details.

### Statistical analysis

Continuous variables are showed as mean ± standard deviation (SD) and categorical variables are expressed as absolute frequency and percentage, *n* (%).

The analysis was to assess whether clinimetric indicators (sensitivity, specificity, accuracy, positive predictive value (PPV), and negative predictive value (NPV) vary detectable as a function of patients' therapeutic status, temporal status, and treatment withdrawal time.

All clinimetric indicators were calculated by SAS routine and the comparison between the proportions was evaluated by z-test on proportions of two independent samples.

Statistical analysis was performed using SAS version 9.4 TS Level 1 M8 (SAS Institute, Cary, NC, USA).

A *p* value < 0.05 was considered statistically detectable.

## Results

Between May 2014 to September 2025, a total of 1016 exams were performed at our Unit.

Of these, 532 were excluded from the analysis due to insufficient clinical or microbiological follow-up.

Final diagnosis was available in 484 patients who were included in this study (Table 1). In 332 patients final diagnosis was based by clinical follow-up obtained through telephone interview conducted 12–24 months after the scintigraphy and by the availability of microbiological data in the remaining 152 cases. Among this group, 114 patients (75%) had a positive culture for pathogenic microorganisms, while 38 (25%) had a negative microbiological finding. No relapses of infection were observed during follow-up.

**Table 1:** General characteristics of studied population

Parameter	*n* (%)
All patients	484 (100)
Male	253 (52.2)
Female	231 (47.8)
Age, mean ± SD, (years)	65.4 ± 14.2
Diagnosis	
Clinical	332 (68.6)
Microbiology	152 (31.4)
*Positive*	*114 (75.0)*
*Negative*	*38 (25.0)*
Osteoarticular	431 (89.0)
*Osteomyelitis*	*86 (19.9)*
*Hip prosthesis*	*116 (26.9)*
*Knee prosthesis*	*168 (39.0)*
*Shoulder prosthesis*	*15 (3.5)*
*Diabetic foot*	*46 (10.7)*
Cardiovascular	42 (8.7)
*Endocarditis*	*3 (7.1)*
*Vascular grafts*	*39 (92.9)*
Soft tissues	11 (2.3)
Antibiotic therapy	
Current antibiotic therapy	64 (13.2)
Therapy discontinued within 7 days	33 (6.8)
Therapy discontinued between 8 and 15 days	17 (3.5)
Therapy discontinued between 16 and 30 days	45 (9.3)
Therapy discontinued for more than 30 days	26 (5.4)
No antibiotic therapy	299 (61.8)
Symptoms	
Symptoms for < 3 months	56 (27.1)
Symptoms between 3 and 12 months	68 (32.9)
Symptoms for > 12 months	83 (40.1)
Time from surgery	
Surgery from < 3 months	35 (16.5)
Surgery between 3 and 12 months	54 (25.5)
Surgery from > 12 months	123 (58)
WBC scintigraphy	
Positive	119 (24.6)
Negative	365 (75.4)

Overall, most frequently isolated microorganisms were Staphylococcus Aureus and Pseudomonas Aeruginosa, followed by Staphylococcus Epidermidis and Escherichia coli (Table 2)

**Table 2:** Isolated bacteria in 114 patients with positive culture

Microrganism	*n*. of patients (% of positives)
*Staphylococcus Aureus*	*43 (37.5)*
*Pseudomonas Aeruginosa*	*19 (16.5)*
*Staphylococcus Epidermidis*	*9 (7.8)*
*Escherichia Coli*	*9 (7.8)*
*Staphylococcus Aureus methicillin-resistant*	*4 (3.5)*
Undefined species	4 (3.5)
Multiple bacteria	15 (13.0)
Other microorganisms	12 (10.4)

WBC count, ESR, CRP, PCT and information on symptoms duration and time from surgery were available in 306 patients and were, therefore, analyzed (Table 3)

We observed high specificity, but low sensitivity for laboratory parameters when considering a cut-off of 11,000 cells for WBC count, 20 mm/h for ESR, 5 mg/L for CRP and 0.05 ng/mL for PCT. Cardiovascular and orthopedic infections showed higher accuracy than soft tissue infections, although significance was not achieved, possibly due to the low number of cases in this last group. Duration of symptoms and time elapsed from surgery did not show any significant influence on the diagnostic accuracy of WBC scintigraphy

**Table 3:** Analysis of whole population and stratified by different laboratory and clinical parameters

Parameter	Sensitivity	Specificity	Accuracy	PPV	NPV	TP	TN	FP	FN
WBC count ≥ 11 × 10^9^/L	60.0	100	73.9	100	57.1	9	8	0	6
ESR ≥ 20 mm/h	56.1	97.1	80.9	92.5	77.3	37	99	3	29
C-reactive protein ≥ 5 mg/L	55.8	96.6	79.4	92.3	75.0	24	57	2	19
PCT > 0.05 ng/ml	60.0	100	79.7	100	70.7	18	29	0	12
Cardiovascular	57.9	100	80.9	100	74.2	11	23	0	8
Osteomyelitis	66.7	96.6	87.0	90.2	86.0	92	283	10	46
Soft tissue	71.4	75.0	72.7	83.3	60.0	5	3	1	2
Symptoms for < 3 months	63.6	100	85.7	100	80.9	14	34	0	8
Symptoms between 3 and 12 months	66.7	100	91.2	100	89.3	12	50	0	6
Symptoms for > 12 months	74.1	96.4	89.2	90.9	88.5	20	54	2	7
Surgery from < 3 months	76.9	95.4	88.6	90.9	87.5	10	21	1	3
Surgery between 3 and 12 months	85.7	–	22.2	23.1	–	12	0	40	2
Surgery from > 12 months	73.7	97.6	90.2	93.3	89.2	28	83	2	10
All patients	65.8	96.6	86.2	90.8	84.7	108	309	11	56

Data for Positive Predictive Value (*PPV*) and Negative Predictive Value (*NPV*) are presented in percentage. Data for True Positive (*TP*), True Negative (*TN*), False Positive (*FP*) and False Negative patients are presented in absolute frequencies

*WBC* White Blood Cell, *ESR* Erythrocyte Sedimentation Rate

The administered activity of ^99m^Tc-HMPAO-WBCs per patient was 736.3 ± 132.5 MBq (19.9 ± 3.6 mCi).

Overall, the labelled leukocyte scintigraphy was positive in 119 patients (24.6%) and negative in 365 patients (75.4%). Out of 119 patients with positive WBC scintigraphy, 66 had a positive microbiology (none with a negative microbiology) and 53 had a positive follow-up and were treated with antibiotic therapy followed or not by surgery depending on clinical cases.

Sensitivity was markedly reduced in patients receiving antibiotics at the time of testing (30%) and increased according to therapy withdrawal time: 50% in the 7 days group, 23% in the 8–15 days group, 100% in the 16–30 days group, 100% in patients with withdrawal > 30 days and 97% in patients without antibiotic therapy. A similar pattern, with progressive improvement at longer withdrawal times, was observed for accuracy and NPV, while specificity and PPV remained stably high in all groups.

Comparative analysis of the 6 patients’ groups categorized for antibiotic therapy showed no significant differences in any of the considered parameters among groups 4, 5 and 6 (supplementary Table 1 and 2). Therefore, these groups were combined and re-categorized as patients “without antibiotic therapy or suspended for > 15 days before WBC scintigraphy” (Group A = group 4 + group 5 + group 6). Similarly, no significant differences were observed between groups 1, 2 and 3, which were re-grouped and classified as patients “with ongoing antibiotic therapy or suspended for ≤ 15 days before WBC scintigraphy” (Group B = group 1 + group 2 + group 3).

**Fig. 1:**
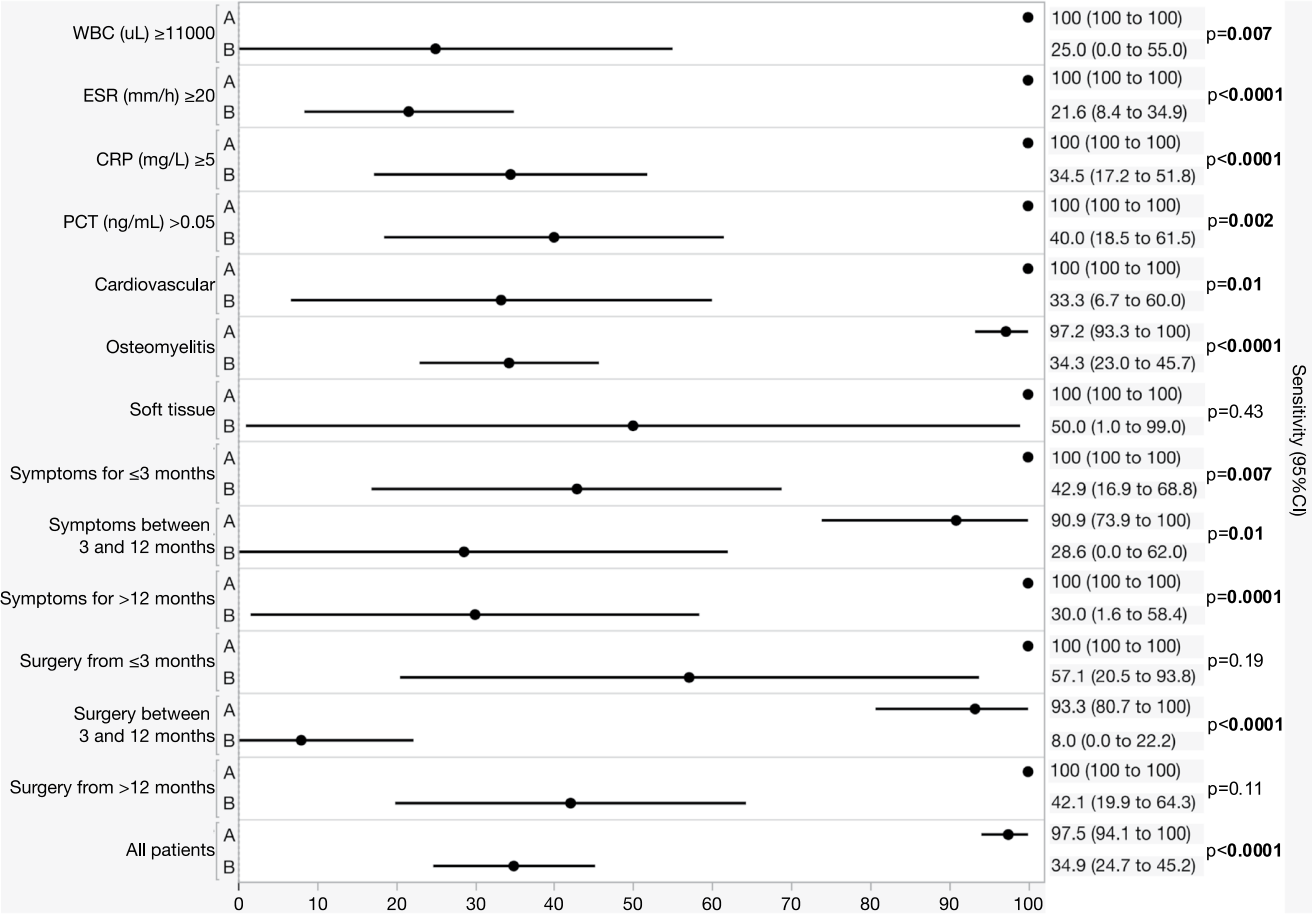
Sensitivity of radiolabelled WBC scintigraphy in whole cohort and stratified by laboratory and clinical parameters

**Fig. 2:**
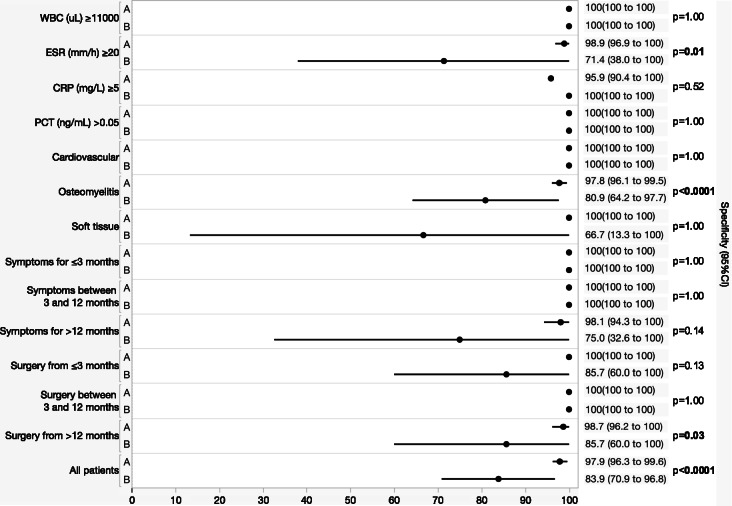
Specificity of radiolabelled WBC scintigraphy in whole cohort and stratified by laboratory and clinical parameters

**Fig. 3:**
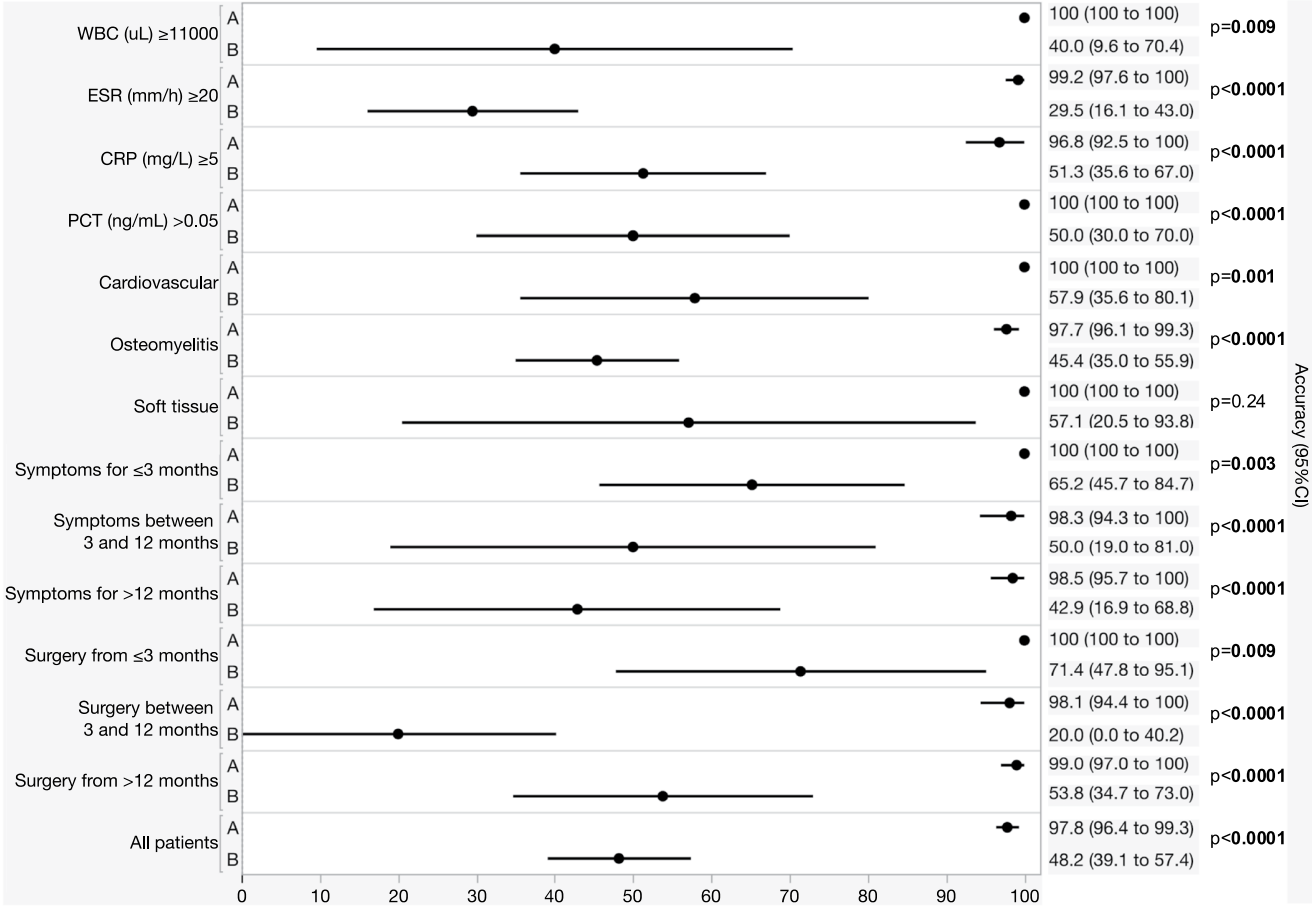
Accuracy of radiolabelled WBC scintigraphy in whole cohort and stratified by laboratory and clinical parameters

**Fig. 4:**
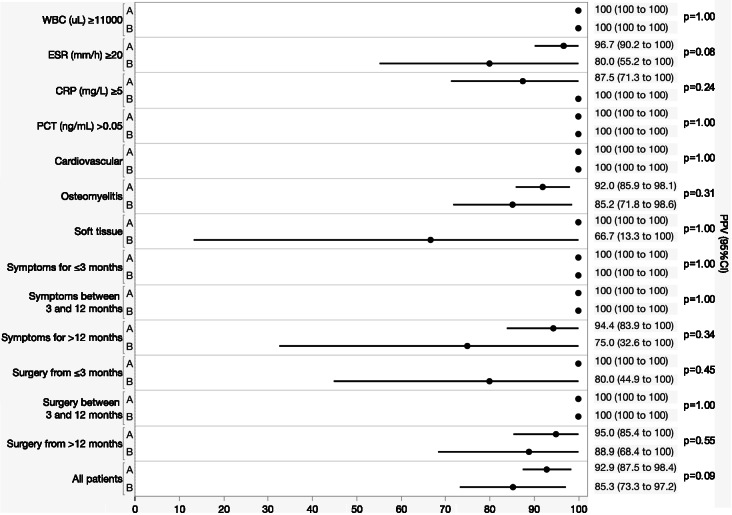
Positive Predictive Value (PPV) of radiolabelled WBC scintigraphy in whole cohort and stratified by laboratory and clinical parameters

**Fig. 5:**
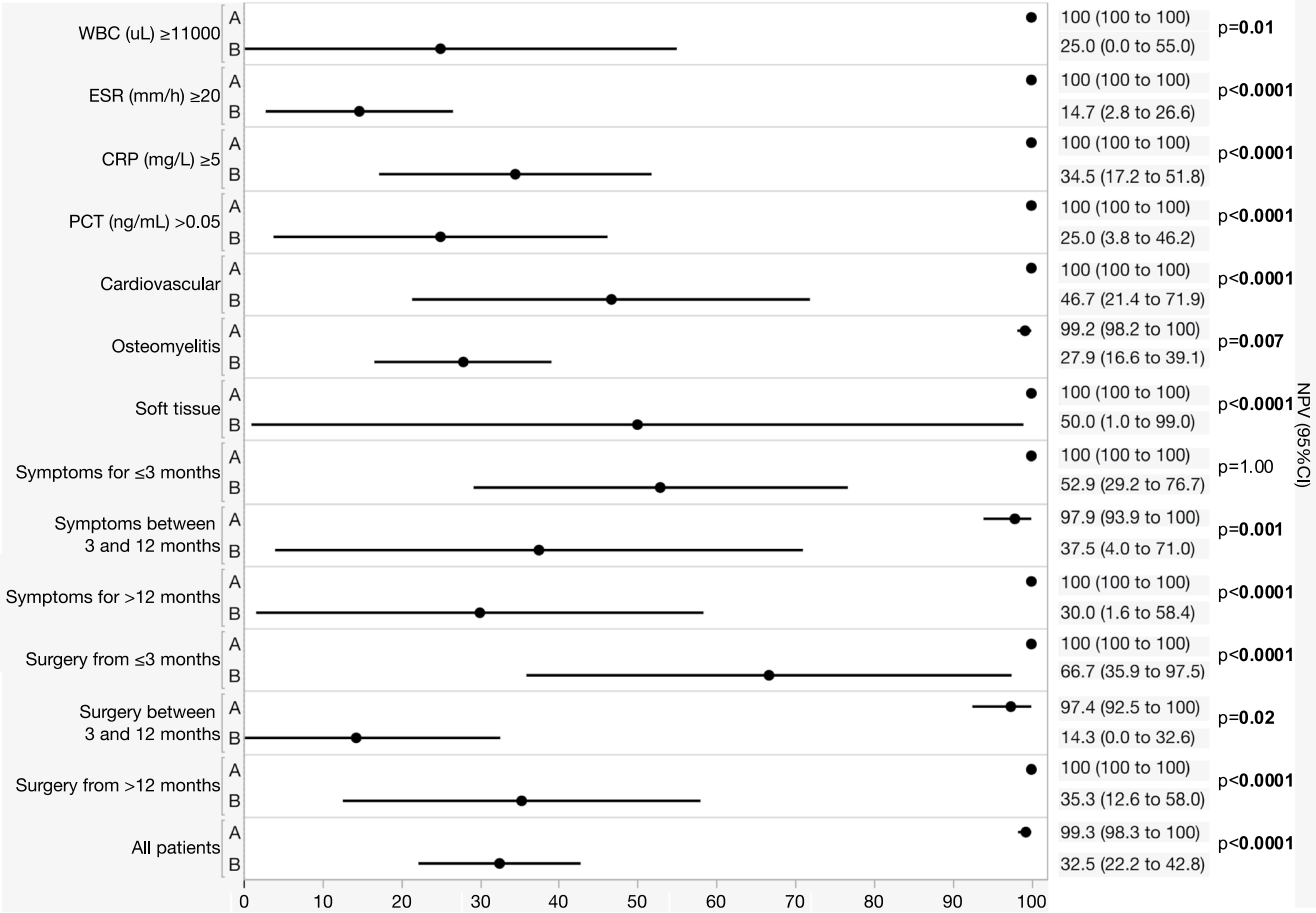
Negative Predictive Value (NPV) of radiolabelled WBC scintigraphy in whole cohort and stratified by laboratory and clinical parameters

Comparison of these two macro-groups showed statistically significant differences of diagnostic accuracy and NPV for all categories (Table 4), but not for soft tissue infections, likely due to the small sample size and large confidence intervals as shown in Figs. 1, 2, 3, 4 and 5.

**Table 4:** Comparison between patients without antibiotic therapy or suspended > 15 days before WBC scintigraphy (group A) and patients with ongoing antibiotic therapy or suspended < 15 days (group B)

Parameter	A									B									A vs. B Sensitivity *p*	A vs. B Specificity *p*	A vs. B Accuracy *p*	A vs. B PPV *p*	A vs. B NPV *p*
	Sensitivity	Specificity	Accuracy	PPV	NPV	TP	TN	FP	FN	Sensitivity	Specificity	Accuracy	PPV	NPV	TP	TN	FP	FN					
WBC count ≥ 11 × 10^9^/L	100 (100 to 100)	100 (100 to 100)	100 (100 to 100)	100 (100 to 100)	100 (100 to 100)	7	6	0	0	25.0 (0.0 to 55.0)	100 (100 to 100)	40.0 (9.6 to 70.4)	100 (100 to 100)	25.0 (0.0 to 55.0)	2	2	0	6	**0.007**	1.00	**0.009**	1.00	**0.01**
ESR ≥ 20 mm/h	100 (100 to 100)	98.9 (96.9 to 100)	99.2 (97.6 to 100)	96.7 (90.2 to 100)	100 (100 to 100)	29	94	1	0	21.6 (8.4 to 34.9)	71.4 (38.0 to 100)	29.5 (16.1 to 43.0)	80.0 (55.2 to 100)	14.7 (2.8 to 26.6)	8	5	2	29	** < 0.0001**	**0.01**	** < 0.0001**	0.08	** < 0.0001**
CRP ≥ 5 mg/L	100 (100 to 100)	95.9 (90.4 to 100)	96.8 (92.5 to 100)	87.5 (71.3 to 100)	100 (100 to 100)	14	47	2	0	34.5 (17.2 to 51.8)	100 (100 to 100)	51.3 (35.6 to 67.0)	100 (100 to 100)	34.5 (17.2 to 51.8)	10	10	0	19	** < 0.001**	0.52	** < 0.0001**	0.24	** < 0.0001**
PCT > 0.05 ng/L	100 (100 to 100)	100 (100 to 100)	100 (100 to 100)	100 (100 to 100)	100 (100 to 100)	10	25	0	0	40.0 (18.5 to 61.5)	100 (100 to 100)	50.0 (30.0 to 70.0)	100 (100 to 100)	25.0 (3.8 to 46.2)	8	4	0	12	**0.002**	1.00	** < 0.0001**	1.00	** < 0.0001**
Cardiovascular	100 (100 to 100)	100 (100 to 100)	100 (100 to 100)	100 (100 to 100)	100 (100 to 100)	7	16	0	0	33.3 (6.7 to 60.0)	100 (100 to 100)	57.9 (35.6 to 80.1)	100 (100 to 100)	46.7 (21.4 to 71.9)	4	7	0	8	**0.01**	1.00	**0.001**	1.00	**0.007**
Osteomyelitis	97.2 (93.3 to 100)	97.8 (96.1 to 99.5)	97.7 (96.1 to 99.3)	92.0 (85.9 to 98.1)	99.2 (98.2 to 100)	69	266	6	2	34.3 (23.0 to 45.7)	80.9 (64.2 to 97.7)	45.4 (35.0 to 55.9)	85.2 (71.8 to 98.6)	27.9 (16.6 to 39.1)	23	17	4	44	** < 0.001**	** < 0.0001**	** < 0.0001**	0.31	** < 0.0001**
Soft tissue	100 (100 to 100)	100 (100 to 100)	100 (100 to 100)	100 (100 to 100)	100 (100 to 100)	3	1	0	0	50.0 (1.0 to 99.0)	66.7 (13.3 to 100)	57.1 (20.5 to 93.8)	66.7 (13.3 to 100)	50.0 (1.0 to 99.0)	2	2	1	2	0.43	1.00	0.24	1.00	1.00
Symptoms for < 3 months	100 (100 to 100)	100 (100 to 100)	100 (100 to 100)	100 (100 to 100)	100 (100 to 100)	8	25	0	0	42.9 (16.9 to 68.8)	100 (100 to 100)	65.2 (45.7 to 84.7)	100 (100 to 100)	52.9 (29.2 to 76.7)	6	9	0	8	**0.007**	1.00	**0.003**	1.00	**0.001**
Symptoms between 3 and 12 months	90.9 (73.9 to 100)	100 (100 to 100)	98.3 (94.3 to 100)	100 (100 to 100)	97.9 (93.9 to 100)	10	47	0	1	28.6 (0.0 to 62.0)	100 (100 to 100)	50.0 (19.0 to 81.0)	100 (100 to 100)	37.5 (4.0 to 71.0)	2	3	0	5	**0.01**	1.00	** < 0.0001**	1.00	** < 0.0001**
Symptoms for > 12 months	100 (100 to 100)	98.1 (94.3 to 100)	98.5 (95.7 to 100)	94.4 (83.9 to 100)	100 (100 to 100)	17	51	1	0	30.0 (1.6 to 58.4)	75.0 (32.6 to 100)	42.9 (16.9 to 68.8)	75.0 (32.6 to 100)	30.0 (1.6 to 58.4)	3	3	1	7	**0.001**	0.14	** < 0.0001**	0.34	** < 0.0001**
Surgery from < 3 months	100 (100 to 100)	100 (100 to 100)	100 (100 to 100)	100 (100 to 100)	100 (100 to 100)	6	15	0	0	57.1 (20.5 to 93.8)	85.7 (60.0 to 100)	71.4 (47.8 to 95.1)	80.0 (44.9 to 100)	66.7 (35.9 to 97.5)	4	6	1	3	0.19	0.13	**0.009**	0.45	**0.02**
Surgery between 3 and 12 months	93.3 (80.7 to 100)	100 (100 to 100)	98.1 (94.4 to 100)	100 (100 to 100)	97.4 (92.5 to 100)	14	38	0	1	8.0 (0.0 to 22.2)	100 (100 to 100)	20.0 (0.0 to 40.2)	100 (100 to 100)	14.3 (0.0 to 32.6)	1	2	0	12	** < 0.0001**	1.00	** < 0.0001**	1.00	** < 0.0001**
Surgery from > 12 months	100 (100 to 100)	98.7 (96.2 to 100)	99.0 (97.0 to 100)	95.0 (85.4 to 100)	100 (100 to 100)	19	77	1	0	42.1 (19.9 to 64.3)	85.7 (60.0 to 100)	53.8 (34.7 to 73.0)	88.9 (68.4 to 100)	35.3 (12.6 to 58.0)	8	6	1	11	0.11	**0.03**	** < 0.0001**	0.55	** < 0.0001**
All patients	97.5 (94.1 to 100)	97.9 (96.3 to 99.6)	97.8 (96.4 to 99.3)	92.9 (87.5 to 98.4)	99.3 (98.3 to 100)	79	283	6	2	34.9 (24.7 to 45.2)	83.9 (70.9 to 96.8)	48.2 (39.1 to 57.4)	85.3 (73.3 to 97.2)	32.5 (22.2 to 42.8)	29	26	5	54	** < 0.0001**	** < 0.0001**	** < 0.0001**	0.09	** < 0.0001**

**A**: Patients without antibiotic therapy or suspended for > 15 days before WBC scintigraphy

**B**: Patients with antibiotic therapy or suspended for ≤ 15 days before WBC scintigraphy

A stratified analysis by antibiotic class was not performed because the active ingredient was available only in a minority of patients and there were significant gaps in the clinical documentation, preventing a reliable and systematic classification.

## Discussion

To the best of our knowledge, this is the largest available study evaluating the impact of ongoing or recently suspended antibiotic therapy on the diagnostic accuracy of labelled leukocyte scintigraphy in the main clinical indications, while comparing its performance across different antibiotic withdrawal timeframes.

One of the first clinical studies, conducted by Datz and Thorne at the end of’80 on patients undergoing 111In-WBC scintigraphy, showed no statistically significant differences in sensitivity between patients receiving antibiotics and untreated patients (88.7% vs. 92.1%), concluding that therapy did not significantly affect test performance [15]. More recent studies, mainly focused on cardiovascular infections (endocarditis, infections of prosthetic valves or cardiac devices), have however re-launched the topic, reporting possible FN in treated patients. It has been hypothesized that prolonged antibiotic therapy may attenuate leukocyte recruitment, especially in low-grade infections or with poorly “leukocyte-attractive” microorganisms [17, 18, 20], thus FN results depend not only on the duration of treatment, but also on the type of infection and class of antibiotics [21]. From a pathophysiological perspective, antibiotic therapy can interfere with labelled WBC by reducing bacterial load and consequently chemotactic gradients, by impairing other neutrophils’ function (phagocytosis and oxidative burst) through several mechanisms, including immunomodulatory effects and alterations in mitochondrial function [14, 22–25].

Our studied population, a large and representative sample from a real-world clinical setting, primarily presented with osteoarticular infections (approximately 89%), reflecting the WBC scan’s role as a reference test in osteomyelitis, prosthetic infections, and diabetic foot [1].

The combination of clinical and microbiological follow-up allowed for robust diagnostic validation. The rate of 31.4% of patients with microbiological confirmation represents a high value for retrospective nuclear medicine studies, where microbiological verification is often incomplete or unavailable [16]. In our sample, approximately 75% of cultures were positive, which is consistent with previous studies on WBC in chronic or subacute infection settings [16, 19].

The distribution of microorganisms confirms the predominance of Gram-positive pathogens like Staphylococcus Aureus, followed by Pseudomonas Aeruginosa and Staphylococcus Epidermidis, mirrors the typical etiology of osteoarticular and prosthetic infections [19]. The presence of polymicrobial infections and unidentified species highlights the diagnostic complexity and the need to integrate microbiological data with imaging data.

While most patients (67.2%) were not under antibiotic treatment or had discontinued them > 30 days, 32.8% of patients were actively taking antimicrobials or had recently stopped antibiotics (≤ 30 days). This heterogeneity reflects real-world clinical practice, in which the need to promptly initiate empirical treatment often prevails over the option of waiting for imaging.

Serum inflammatory markers, available in a small portion of patients, suggest that our population was predominantly affected by chronic or subacute infections with moderate or absent elevations of inflammatory markers and low-bacterial burden or localized infections [1, 16]. Although sensitive in the acute phases, they do not correlate linearly with scintigraphic positivity in chronic or localized infectious processes as previously demonstrated [16, 19].

Our results confirm that inflammatory markers represent a useful clinical aid in selecting patients for imaging, but they are not a substitute for WBC scintigraphy.

Overall, we observed a sensitivity of 66%, a specificity of 97% and 86% of accuracy in line with meta-analyses and retrospective studies [26–28].

Diagnostic performance was significantly affected by antibiotic withdrawal timing, with markedly reduced sensitivity in patients receiving therapy at the time of the test and values ​​progressively improved in the groups with increasing withdrawal time. The best performance was achieved in patients who were not receiving treatment or who had stopped treatment for more than 15 days.

Therefore, the trend observed in our study—very low sensitivity during ongoing therapy, progressive improvement after short suspension (7 days) and near-optimal sensitivity ​​after 16–30 days and beyond- may be partially explained by the immune system’s re-gained ability to recruit leukocytes into the infectious site.

Another interesting finding of our study is the absence of significant differences in PPV between the two macro-groups (Fig. 4). This suggests that a positive WBC scintigraphy retains high diagnostic confidence, irrespective of antibiotic discontinuation. Conversely, the NPV, which reflects the test's ability to accurately rule out the disease, is more affected by the increase in false negatives observed during ongoing therapy or recent discontinuation (Fig. 5). Thus, antibiotic use primarily impacts WBC scintigraphy’s ability to exclude an infection, rather to confirm it.

This study has several limitations. First of all, its retrospective design implies that some clinical and laboratory data are incomplete, including the type and duration of antibiotic therapy, limiting deeper analyses. The duration of antibiotic therapy might have different impact on leukocyte migration and homing in infected tissues and therefore could impact on the performance of WBC scan. However, information on antibiotics duration was available only for few patients of our cohort. This is an issue that must be addressed by other retrospective and prospective studies.

Moreover, it would be clinically interesting to investigate whether different classes of antibiotics have varying impacts on the performance of WBC scintigraphy, potentially identifying antibiotics that do not interfere with the scan. This could significantly impact patients’ preparation to WBC scan. Unfortunately, due to the retrospective nature, this information was available only in a limited number of patients thus preventing any further speculation.

Further studies are needed to assess differences between antibiotic classes with pronounced immunomodulatory effects, which can impact neutrophil chemotaxis, phagocytosis, and oxidative burst with potential repercussions on the interpretation of WBC scintigraphy. (e.g. macrolides, quinolones, and tetracyclines), and those generally considered "neutrophil-sparing" (e.g. ß-lactams, glycopeptides, aminoglycosides and fosfomycin).

From a methodological point of view, the uneven distribution of patients across the groups (with fewer patients in the 10–15 and 16–30 days groups) warrants careful interpretation of the results, especially for comparisons approaching statistical significance. Moreover, combining together group 1, 2 and 3 and groups 4, 5 and 6 together might not be ideal from a purely clinical perspective, as these groups represent distinct clinical scenarios and different pre-test probabilities. However, since we did not observe significant differences in any of the considered parameters we decided to re-categorize as patients “without antibiotic therapy or suspended for > 15 days before WBC scintigraphy” (Group A), and patients “with ongoing antibiotic therapy or suspended for ≤ 15 days before WBC scintigraphy” (Group B).

Another limitation is the lack of a formal distinction between the type of infection, anatomical site, and the causative microorganism (e.g., Gram-positive vs. Gram-negative, bacteria with reduced leukocyte recruitment capacity such as some *Enterococcus spp.*, or opportunistic pathogens such as *Candida spp.*), which can influence the sensitivity of labelled leukocyte scintigraphy [12, 13].

Moreover, final diagnosis in a significant portion of patients was achieved through clinical follow-up, rather than definitive microbiological confirmation. While this approach is common in clinical practice, this might have led to an overestimation or underestimation of the prevalence of the infection in our population.

Nevertheless, despite these inherent limitations, this study is the first to stratify antibiotic discontinuation into distinct time windows, highlighting a clear gradient of improvement in diagnostic accuracy with increasing washout period. This suggests that a negative WBC scintigraphy in patients undergoing or recently completing antibiotic therapy should be interpreted with caution. Based on our results, 15 days of suspension are advisable to perform WBC scintigraphy, but this warrants further investigations.

## Conclusions

Results of this study indicate that the accuracy of radiolabelled WBC scintigraphy is significantly influenced by the presence and timing of antibiotic therapy. Performing the test during antibiotic treatment is associated with a substantial reduction in sensitivity and NPV, increasing false negatives and compromising its ability to rule out infection. Conversely, gradual withdrawal of therapy results in a progressive recovery of leukocyte function, with performance returning to the expected levels in the literature when antibiotic washout exceeds 15 days. Our data highlight the importance of managing antibiotic withdrawal when planning the test to ensure its correct interpretation. Therefore, a collaborative approach with the referring clinician is essential to determine patient’s antibiotic history, current clinical status, ensuring appropriate test interpretation.

## Supplementary Information

Below is the link to the electronic supplementary material.

Supplementary file1 (DOCX 23 KB)

Supplementary file2 (DOCX 22 KB)

## Data Availability

The datasets generated during and/or analysed during the current study are available from the corresponding author on reasonable request.
