# Supplementary material for: Diagnostic accuracy of radiolabelled-WBC scintigraphy in patients with antibiotic therapy
Source: Eur J Nucl Med Mol Imaging. 2026 Apr 29;53(9):5605–16. doi: 10.1007/s00259-026-07855-w (PMC13314914; doi:10.1007/s00259-026-07855-w)
Supplement: Supplementary file 1 — Supplementary file1 (DOCX 23 KB) [file 259_2026_7855_MOESM1_ESM.docx]

**Supplementary table 1**

**Comparison between patients under antibiotic therapy and patients with antibiotic therapy suspended**

**up to 15 days before scintigraphy**

| **Parameter** | **A** | | | **B** | | | **C** | | | **A** vs. **B** Sensitivity p | **A** vs. **B** Specificity p | **A** vs. **B** Accuracy p | **A** vs. **C** Sensitivity p | **A** vs. **C** Specificity p | **A** vs. **C** Accuracy p |
| --- | --- | --- | --- | --- | --- | --- | --- | --- | --- | --- | --- | --- | --- | --- | --- |
|  | **Sensitivity** | **Specificity** | **Accuracy** | **Sensitivity** | **Specificity** | **Accuracy** | **Sensitivity** | **Specificity** | **Accuracy** |  |  |  |  |  |  |
| **WBC count ≥ 11 x 10^9^/L** | 28.6 | 100 | 44.4 | - | - | - | - | - | - | - | - | - | - | - | - |
| **ESR ≥ 20 mm/h** | 13.6 | 50.0 | 16.7 | 40.0 | 66.7 | 46.1 | 20.0 | 100 | 42.9 | 0.16 | 1.00 | 0.12 | 1.00 | 1.00 | 0.30 |
| **C-reactive protein ≥ 5 mg/L** | 41.2 | 100 | 56.5 | 50.0 | 100 | 57.1 | - | 100 | 33.3 | 1.00 | 1.00 | 1.00 | - | 1.00 | 0.43 |
| **Procalcitonin > 0.05 ng/L** | 46.1 | 100 | 58.8 | 63.7 | - | 63.7 | 33.3 | - | 33.3 | 1.00 | - | 1.00 | 1.00 | - | 0.57 |
| **Cardiovascular** | 28.6 | 100 | 58.3 | 33.3 | 100 | 60.0 | 50.0 | - | 50.0 | 1.00 | 1.00 | 1.00 | 1.00 | - | 1.00 |
| **Osteomyelitis** | 28.9 | 70.0 | 37.5 | 55.5 | 85.7 | 64.0 | 18.2 | 100 | 40.0 | 0.08 | 0.60 | 0.051 | 0.70 | 0.51 | 1.00 |
| **Soft tissue** | 100 | 66.7 | 75 | 33.3 | - | 33.3 | - | - | - | 1.00 | - | 0.49 | - | - | - |
| **Symptoms for <3 months** | 27.3 | 100 | 55.5 | 100 | 100 | 100 | - | - | - | 0.055 | 1.00 | 0.12 | - | - | - |
| **Symptoms between 3 and 12 months** | 50.0 | 50.0 | 50 | 100 | 100 | 100 | - | 100 | 33.3 | 0.46 | 1.00 | 0.24 | - | 1.00 | 1.00 |
| **Symptoms for >12 months** | 20.0 | - | 16.7 | 33.3 | 100 | 50.0 | 50.0 | 100 | 75.0 | 1.00 | - | 0.50 | - | 0.33 | 0.19 |
| **Surgery from <3 months** | 40.0 | 80.0 | 60 | 100 | 100 | 100 | - | - | - | 0.43 | 1.00 | 0.25 | - | - | - |
| **Surgery between 3 and 12 months** | 50.0 | 50.0 | 50 | - | - | - | - | - | - | - | - | - | - | - | - |
| **Surgery from >12 months** | 22.2 | 50.0 | 27.3 | 71.4 | 100 | 77.8 | 33.3 | 100 | 66.7 | 0.13 | 1.00 | 0.07 | 1.00 | 0.40 | 0.16 |
| **All patients** | 30.4 | 77.8 | 43.7 | 50.0 | 88.9 | 60.6 | 23.1 | 100 | 41.2 | 0.11 | 0.64 | 0.12 | 0.30 | 0.11 | 1.00 |

**A**: Patients under antibiotic therapy; **B**: Patients with antibiotic therapy suspended for ≤ 7 days; **C**: Patients with antibiotic therapy suspended between 8 and 15 days

Data for sensitivity, specificity and accuracy are presented in percentage.

WBC=white blood cell; ESR=erythrocyte sedimentation rate.
