# Supplementary material for: Diagnostic accuracy of radiolabelled-WBC scintigraphy in patients with antibiotic therapy
Source: Eur J Nucl Med Mol Imaging. 2026 Apr 29;53(9):5605–16. doi: 10.1007/s00259-026-07855-w (PMC13314914; doi:10.1007/s00259-026-07855-w)
Supplement: Supplementary file 2 — Supplementary file2 (DOCX 22 KB) [file 259_2026_7855_MOESM2_ESM.docx]

**Supplementary table 2**

**Comparison between patients without antibiotic therapy and patients who suspended antibiotic therapy**

**for 16 days or more before scintigraphy**

| **Parameter** | **A** | | | **B** | | | **C** | | | **A** vs. **B** Sensitivity p | **A** vs. **B** Specificity p | **A** vs. **B** Accuracy p | **A** vs. **C** Sensitivity p | **A** vs. **C** Specificity p | **A** vs. **C** Accuracy p |
| --- | --- | --- | --- | --- | --- | --- | --- | --- | --- | --- | --- | --- | --- | --- | --- |
|  | **Sensitivity** | **Specificity** | **Accuracy** | **Sensitivity** | **Specificity** | **Accuracy** | **Sensitivity** | **Specificity** | **Accuracy** |  |  |  |  |  |  |
| **WBC count ≥ 11000** | 100 | 100 | 100 | - | 50.0 | 50.0 | - | - | - | - | 0.09 | 0.20 | - | - | - |
| **ESR ≥ 20** | 100 | 100 | 100 | 100 | 93.3 | 96.0 | 100 | 100 | 100 | 1.00 | 0.06 | 0.06 | 1.00 | 1.00 | 1.00 |
| **C-reactive protein ≥ 5** | 100 | 97.4 | 97.9 | 100 | 88.9 | 92.9 | - | 100 | 100 | 1.00 | 0.26 | 0.35 | - | 1.00 | 1.00 |
| **Procalcitonin > 0.05** | 100 | 100 | 100 | 100 | 100 | 100 | - | 100 | 100 | 1.00 | 1.00 | 1.00 | 1.00 | 1.00 | 1.00 |
| **Cardiovascular** | 100 | 100 | 100 | 100 | - | 100 | - | 100 | 100 | 1.00 | - | 1.00 | - | 1.00 | 1.00 |
| **Osteomyelitis** | 96.1 | 97.8 | 97.5 | 100 | 96.3 | 97.7 | 100 | 100 | 100 | 0.42 | 0.64 | 0.93 | 1.00 | 0.49 | 0.43 |
| **Soft tissue** | 100 | 100 | 100 | 100 | - | 100 | - | - | - | 1.00 | - | - | - | - | - |
| **Symptoms for <3 months** | 100 | 100 | 100 | 100 | 100 | 100 | 100 | 100 | 100 | 1.00 | 1.00 | 1.00 | 1.00 | 1.00 | 1.00 |
| **Symptoms between 3 and 12 months** | 85.7 | 100 | 97.4 | 100 | 100 | 100 | - | 100 | 100 | 0.43 | 1.00 | 0.56 | - | 1.00 | 0.69 |
| **Symptoms for >12 months** | 100 | 97.9 | 98.3 | 100 | 100 | 100 | 100 | 100 | 100 | 1.00 | 0.83 | 0.77 | 1.00 | 1.00 | 0.82 |
| **Surgery from <3 months** | 100 | 100 | 100 | 100 | 100 | 100 | - | 100 | 100 | 1.00 | 1.00 | 1.00 | - | 1.00 | 1.00 |
| **Surgery between 3 and 12 months** | 87.5 | 100 | 97.0 | 100 | 100 | 100 | - | 100 | 100 | 0.33 | 1.00 | 0.5 | - | 1.00 | 0.69 |
| **Surgery from >12 months** | 100 | 98.5 | 98.9 | 100 | 100 | 100 | 100 | 100 | 100 | 1.00 | 0.83 | 0.77 | 1.00 | 1.00 | 0.74 |
| **All patients** | 96.7 | 97.9 | 97.7 | 100 | 96.3 | 97.8 | 100 | 100 | 100 | 0.43 | 0.59 | 1.00 | 1.00 | 0.48 | 0.43 |

**A**: Patients without antibiotic therapy; **B**: Patients with antibiotic therapy suspended between 16 and 30 days; **C**: Patients with antibiotic therapy suspended for >30 days

Data for sensitivity, specificity and accuracy are presented in percentage.

WBC=white blood cell; ESR=erythrocyte sedimentation rate.
